# Synthesis of 2-Aminopyrimidine Derivatives and Their Evaluation as *β*-Glucuronidase Inhibitors: In Vitro and In Silico Studies

**DOI:** 10.3390/molecules27227786

**Published:** 2022-11-11

**Authors:** Sarosh Iqbal, Nimra Naveed Shaikh, Khalid Mohammed Khan, Shumaila Kiran, Sehrish Naz, Zaheer Ul-Haq, Shahnaz Perveen, M. Iqbal Choudhary

**Affiliations:** 1 Department of Applied Chemistry, Government College University, Faisalabad 38000, Pakistan; 2 H. E. J. Research Institute of Chemistry, International Center for Chemical and Biological Sciences, University of Karachi, Karachi 75270, Pakistan; 3 Department of Clinical Pharmacy, Institute for Research and Medical Consultations (IRMC), Imam Abdulrahman Bin Faisal University, Dammam P.O. Box 31441, Saudi Arabia; 4 Dr. Panjwani Center for Molecular Medicine and Drug Research, International Center for Chemical and Biological Sciences, University of Karachi, Karachi 75270, Pakistan; 5 PCSIR Laboratories Complex, Shahrah-e-Dr. Salimuzzaman Siddiqui, Karachi 75280, Pakistan

**Keywords:** 2-Amino-4,6-dichloropyrimidine, nucleophilic substitution reaction, catalyst-free synthesis, solvent-free synthesis, *β*-glucuronidase inhibitors, structure-activity relationship, in silico study

## Abstract

Currently the discovery and development of potent *β*-glucuronidase inhibitors is an active area of research due to the observation that increased activity of this enzyme is associated with many pathological conditions, such as colon cancer, renal diseases, and infections of the urinary tract. In this study, twenty-seven 2-aminopyrimidine derivatives **1–27** were synthesized by fusion of 2-amino-4,6-dichloropyrimidine with a variety of amines in the presence of triethylamine without using any solvent and catalyst, in good to excellent yields. All synthesized compounds were characterized by EI-MS, HREI-MS and NMR spectroscopy. Compounds **1–27** were then evaluated for their *β*-glucuronidase inhibitory activity, and among them, compound **24** (IC_50_ = 2.8 ± 0.10 µM) showed an activity much superior to standard D-saccharic acid 1,4-lactone (IC_50_ = 45.75 ± 2.16 µM). To predict the binding mode of the substrate and *β*-glucuronidase, in silico study was performed. Conclusively, this study has identified a potent *β*-glucuronidase inhibitor that deserves to be further studied for the development of pharmaceutical products.

## 1. Introduction

**Figure 1:**
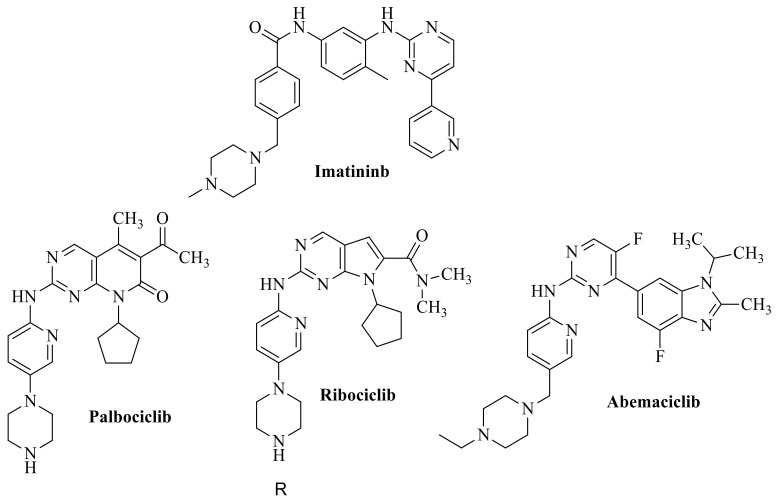
2-Aminopyrimidine derivatives as anticancer drugs.

2-Aminopyrimidines constitute an important class of heterocycles known for diverse activities, such as anticancer, antioxidant, antibacterial, antifungal, antiviral, anti-inflammatory, antimalarial, antidiabetic, antileishmanial, and antitrypanosomal properties [1,2,3,4,5,6]. The 2-aminopyrimidine-containing anticancer drugs, namely imatinib, palbociclib, ribociclib, and abemaciclib, are in use (Figure 1) [7,8]. These 2-aminopyrimidines are also used as a starting material to synthesize other fused heterocycles such as imidazopyrimidines, triazolopyrimidines, pyridopyrimidines, and pyrimidopyrimidines [9,10].

The *β*-glucuronidase enzyme belongs to the glycosidase family that catalyze the breakdown of complex carbohydrates. It is a prime component of phase II detoxification that helps to remove carcinogens, tumor promoters, estrogen, bile acids, and pharmaceuticals from living bodies [11]. It is observed that *β*-glucuronidase enzyme activity is significantly increased in patients that have *β*-glucuronidase-producing bacteria in the intestine. Overexpression of *β*-glucuronidase leads to excessive hydrolysis of glucuronide and liberation of xenobiotics, which ultimately leads to toxicity in the intestine and eventually causes several pathologies [12,13,14,15,16,17,18]. Therefore, inhibition of *β*-glucuronidase activity is an imperative area of research to reduce gastrointestinal toxicity, intestinal disorders, and hormone-dependent cancers, such as breast, prostate, and colonic carcinogenesis.

Currently, there is a major interest in the discovery of natural and synthetic *β*-glucuronidase inhibitors [19,20,21,22,23,24]. Our research group has also reported earlier several heterocycles like oxadiazole, thiadiazole, thiazole, and quinazolines, etc., as promising *β*-glucuronidase inhibitors [25,26,27,28,29,30]. As quinazolines are benzo fused pyrimidines, therefore, we decided to explore *β*-glucuronidase inhibitory potential of pyrimidines. Herein, we report 2-aminopyrimidines as a new class of *β*-glucuronidase inhibitors.

## 2. Results and Discussion

### 2.1. Chemistry

**Scheme 1:**
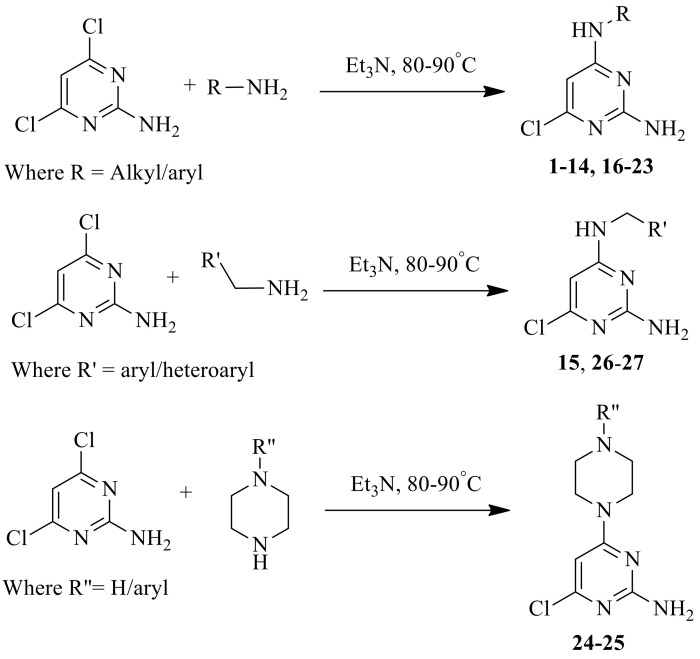
Synthesis of 2-aminopyrimidine derivatives **1–27**.

The 2-aminopyrimidine derivatives **1–27** were synthesized in high yields by using method reported by us previously [31]. Commercially available 2-amino-4,6-dichloropyrimidine reacted with different amines in the presence of triethylamine in solvent-free conditions at 80–90 °C to yield products **1–27** (Scheme 1). The structures of the resulting compounds were deduced by NMR spectroscopy, EI-MS, and HREI-MS spectrometry. All compounds have also furnished satisfactory elemental analyses. Structures of synthesized compounds are presented in Table 1.

### 2.2. Bioassay

#### 2.2.1. In Vitro *β*-Glucuronidase Inhibition Activity

**Figure 2:**
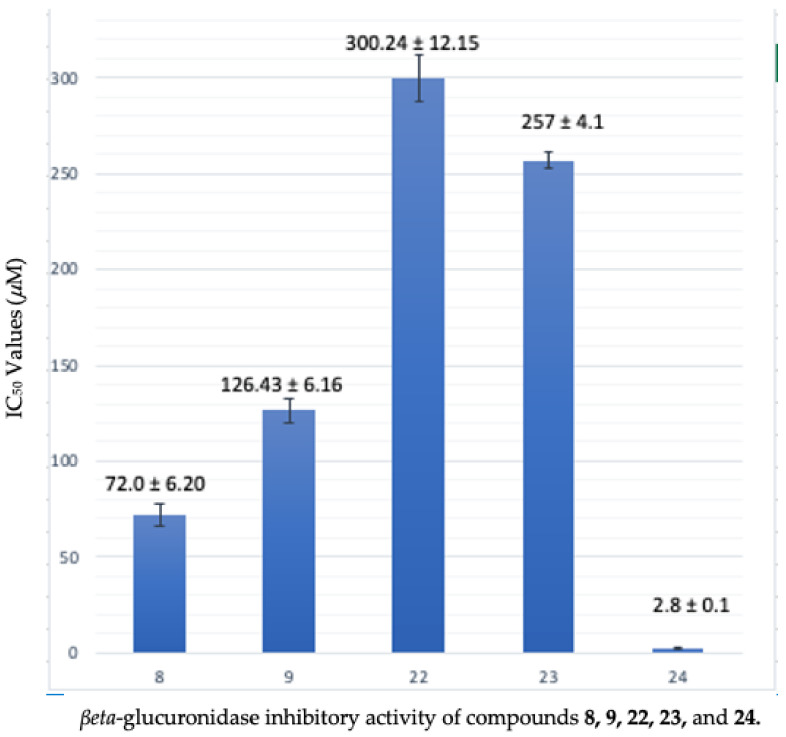
Bar chart diagram for *β*-glucuronidase Inhibitory activity of compounds **1–27**.

Synthetic analogs **1–27** were evaluated for their in vitro *β*-glucuronidase inhibitory activity (Table 2). Among them, five compounds showed varying degrees of enzyme inhibition with IC_50_ values between 2.8 ± 0.10–300.25 ± 12.15 µM. Compound **24** (IC_50_ = 2.8 ± 0.10 µM) showed an activity many times higher than standard D-saccharic acid 1,4- lactone (IC_50_ = 45.75 ± 2.16 µM). Likewise, compounds **8** (IC_50_ = 72.0 ± 6.20 µM) and **9** (IC_50_ = 126.43 ± 6.16 µM) exhibited significant inhibition, whereas compounds **22** (IC_50_ = 300.25 ± 12.5 µM) and **23** (IC_50_ = 257.0 ± 4.18 µM) were only weakly active. Moreover, the rest of the compounds showed less than 50% inhibitory activity at 0.2 mM concentration, and thus were not evaluated for IC_50_ values (Figure 2).

**Figure 3:**
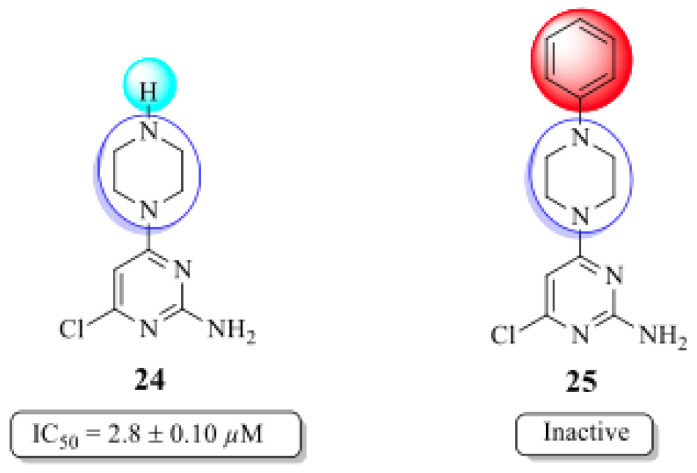
Comparison of in vitro *β*-glucuronidase inhibition potential of compounds having piperazinyl and phenylpiperazinyl substitution at pyrimidine ring.

Compound **24** (IC_50_ = 2.8 ± 0.10 µM), having a piperazinyl substituent at C-4 of pyrimidine ring, was the most potent in vitro *β*-glucuronidase inhibitor. In contrast, compound **25** having 4-phenyl piperazinyl substituent at C-4 of pyrimidine ring was inactive. Therefore, it can be inferred that the hydrogen atom attached to the piperazinyl moiety might be playing an important role for in vitro *β*-glucuronidase inhibitory activity of compound **24** (Figure 3).

**Figure 4:**
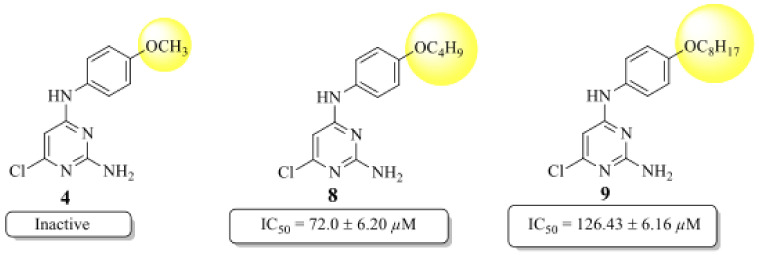
Comparison of *β*-glucuronidase inhibitory activity of compounds with a 4-alkoxy substitution at the benzene ring.

When in vitro *β*-glucuronidase inhibition activity of compounds having alkoxy substitution at C-4 of the phenyl ring were compared, we observed that compound **4** with a methoxy substituent was inactive, whereas compound **8** having butoxy (IC_50_ = 72.0 ± 6.20 µM) and compound **9** (IC_50_ = 126.43 ± 6.16 µM) having octyloxy substituents has shown *β*-glucuronidase inhibitory activity. The results indicated that alkoxy chain length is important for activity (Figure 4).

**Figure 5:**
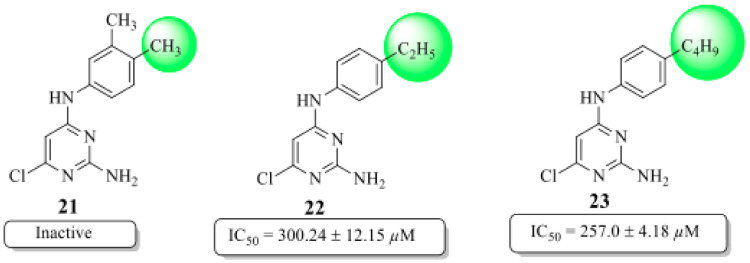
Comparison of *β*-glucuronidase inhibitory activity of compounds with a 4-alkyl substitution at the benzene ring.

Likewise, when we compared the activity of compounds with alkyl substitution at C-4 of the phenyl ring, compound **21** with a methyl substituent was found inactive, whereas compound **22** (IC_50_ = 300.25 ± 12.5 µM) with an ethyl and compound **23** (IC_50_ = 126.43 ± 6.16 µM) with a butyl substituent showed *β*-glucuronidase inhibition. Here, we also observed that alkyl chain length is important for activity. With the increase in chain length, *β*-glucuronidase inhibition activity increases (Figure 5).

#### 2.2.2. In Vitro Urease Inhibition Activity

Synthetic analogs **1–27** were also evaluated for their in vitro urease inhibition potential. All tested compounds showed either no or less than 50% urease inhibition at 0.2 mM concentration, so they were considered inactive against urease enzyme.

#### 2.2.3. Antioxidant Studies

Synthetic derivatives **1–27** were tested for estimating their in vitro 1,1-diphenyl-2-picrylhydrazyl (DPPH) radical scavenging and superoxide scavenging potential, but none of the compounds showed any antioxidant potential at 0.2 mM concentration.

### 2.3. In Silico Studies

**Figure 6:**
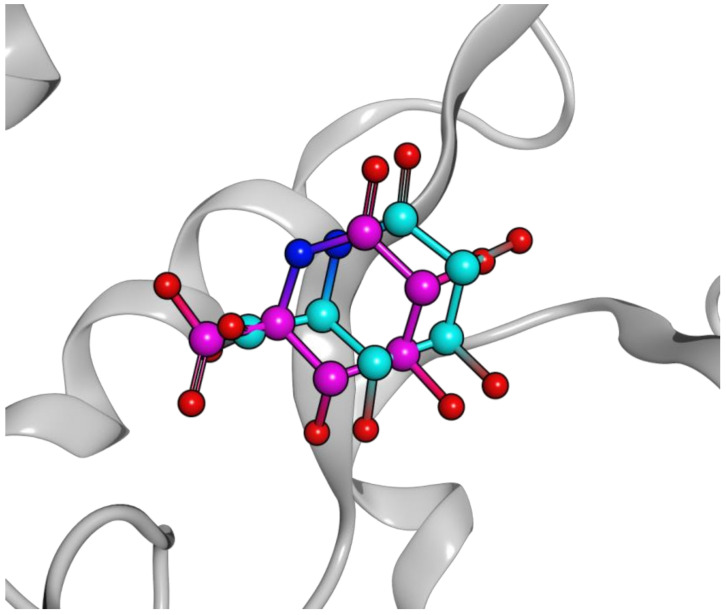
Redock pose (magenta) of the substrate (2*S*,3*R*,4*S*,5*R*)-3,4,5-trihydroxy-6-oxopiperidine-2-carboxylic acid (EVA), superimposed over its crystallized pose (cyan), within the binding pocket of *E. coli β*-glucuronidase.

To predict the binding modes and molecular interactions of newly synthesized 2-aminopyrimidine derivatives, the docking module of MOE was employed. Prior to the docking study, the efficiency and reliability of docking software in terms of our target protein were evaluated by a redocking experiment. For this purpose, the three-dimensional X-ray crystal structure of *β*-glucuronidase from *E. coli* (PDB ID 3K4D), bound to the substrate (2S,3R,4S,5*R*)-3,4,5-trihydroxy-6-oxopiperidine-2-carboxylic acid (EVA), was retrieved from a protein data bank (http://www.rcsb.org/pdb, accessed on 17 October 2022) on 5 February 2022. This co-crystallized substrate compound was extracted and redocked to the binding cavity of the target protein *via* MOE 2013 [32]. A reliable root mean square deviation (RMSD) of 0.4 Å (Figure 6) indicated MOE to be a suitable software to conduct docking studies of compounds **1–27**.

Our docking results showed a good agreement with the experimental data. Most 2-aminopyrimidine derivatives were found to be inactive with very few compounds displaying significant inhibition in both in vitro and in silico studies. Before starting molecular docking, all compounds were divided into four groups based on their in vitro inhibitory activities. Compounds that showed IC_50_ less than 50 µM were categorized in “Group A”, while compounds with moderate activity (IC_50_ = 50–100 µM) were categorized into “Group B”, and the compounds with least activity (IC_50_ g ≥ 100 µM) were classified as “Group C”. The rest of the compounds with no inhibitory activity were placed in “Group D” (Table 2).

**Figure 7:**
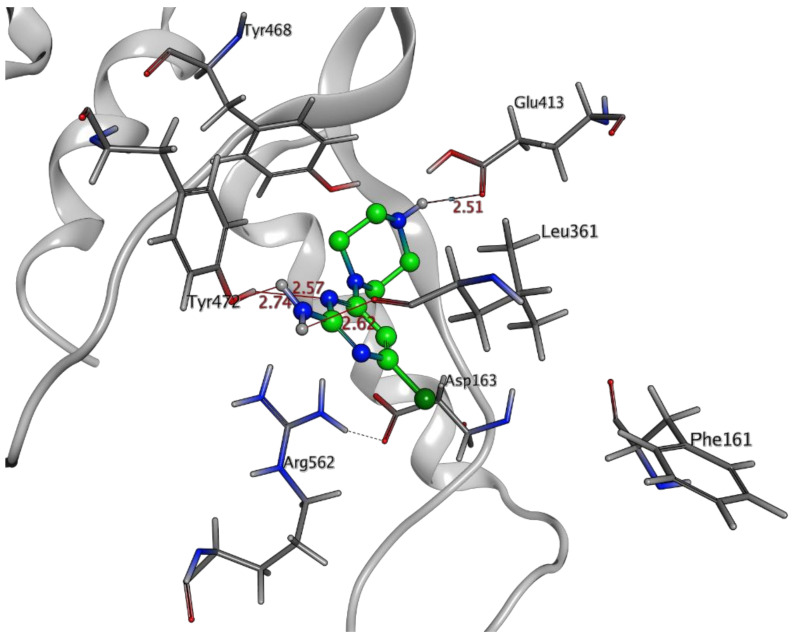
3-Dimensional interaction diagram of the most potent compound **24** (green) presenting various hydrogen bonds (red lines) and hydrophobic interactions with the crucial residues (Glu413 and Leu361) of *β*-glucuronidase responsible for its potent inhibitory activity.

As per observed activities, the active compounds showed noticeable hydrogen bonding and hydrophobic interactions with the hotspot residues of the target protein, i.e., Glu413 and Leu361. Docking analysis further indicated that these derivatives exhibit marked hydrogen bonding with Glu413, along with hydrophobic interactions with Leu361. Among the compounds of group A, compound **24** showed a significant interactions with Glu413 and Leu361 in a similar manner as the reference molecule (EVA) [33]. The most potent inhibitor **24** virtually occupied a similar position as observed by the substrate (EVA) in the crystal structure. Compound **24**, having a piperazinyl substituent at C-4 of pyrimidine ring, was observed to establish good interactions with key residues. The nitrogen of piperazine moiety established two hydrogen bonds, one with the crucial residue Glu413 and one with Tyr468 at a distance of 2.57 Å and 2.44 Å, respectively. While this interaction was not observed in the case of compound **25**. Moreover, the carbonyl group of Leu361 presented hydrogen bonding (2.62 Å) with the amine functionality attached to the pyrimidine ring, while the hydrogen of Leu361 established noticeable hydrophobic interactions with the piperazine ring of compound **24**. Apart from it, two additional hydrogen bonds were observed between the hydroxyl group of Tyr472 and the nitrogen and amine group of the pyrimidine ring of the compound, at a distance of 2.57 Å and 2.74 Å, respectively (Figure 7). Whereas compound **25** having 4-phenylpiperazinyl substituent at C-4 of pyrimidine ring exhibited different orientation within the cavity, and thus unable to form significant interactions with the active site residues which could be the reason of its inactivity.

**Figure 8:**
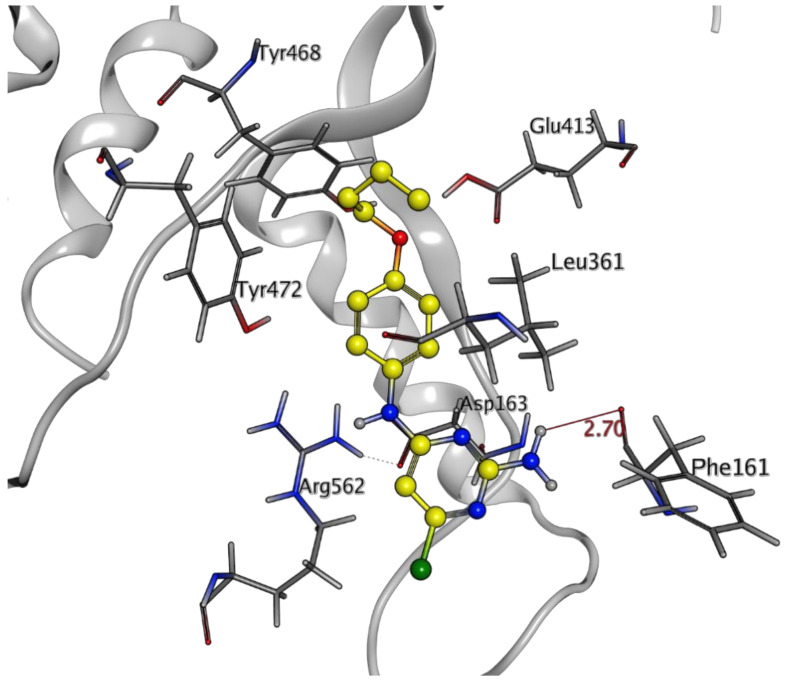
Molecular interaction map of moderate active compound **8** (yellow) displaying very few electrostatic (red line) and hydrophobic interactions with noncrucial residues within the binding pocket of the *β*-glucuronidase enzyme.

**Figure 9:**
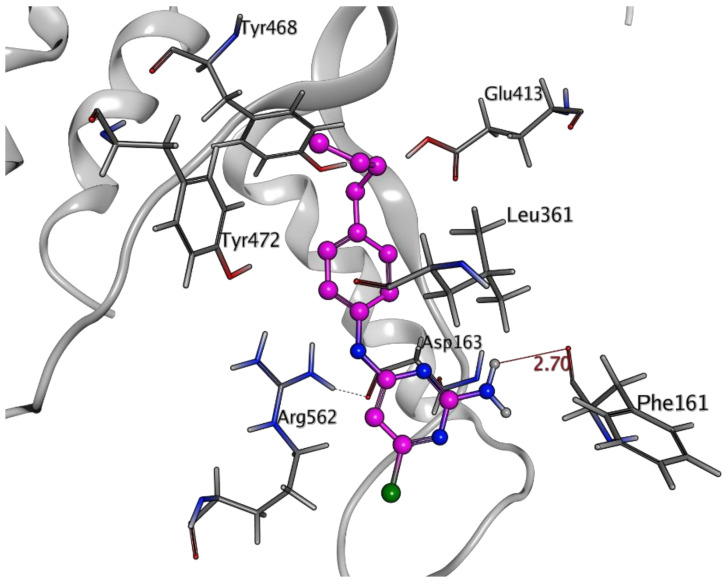
The three-dimensional picture of the least active compound **23** presents the similar binding and molecular interactions, as exhibited by a moderately active compound **8**, with insignificant residues in the binding pocket of *β*-glucuronidase.

The visual inspection of the moderately active compounds **8** and **9** highlighted that the substituted part of these compounds, i.e., the benzene ring with an aliphatic chain, moves deep within the cavity of the target protein. While inactive compound **4,** having a methoxy substituent, was unable to establish interaction with the cavity residues which indicated that length of alkoxy chain is important for in vitro *β*-glucuronidase inhibition. Our docking analysis showed that in compound **8**, the amine group attached to the pyrimidine ring formed a hydrogen bond with the carbonyl of Phe161 at a distance of 2.70 Å and side chain (butoxy) formed hydrophobic interactions with noncrucial residues, like Val446 and Tyr472 (Figure 8). Similarly, visualization of the least active and inactive compounds, such as **21–23** at the molecular level presented the same picture as moderate active compounds. However, due to the difference in aliphatic chain, all three compounds showed different level of binding with the active site, which could be the reason of their differential activity. It was observed that compound **21** with methyl substituent is less stable, as it is not involved in interaction with active site residues. Whereas compound **22** with ethyl and compound **23** with butyl substituents showed interactions with active site residue, responsible for *β*-glucuronidase inhibitory potential. In the case of compound **23**, the amine attached to the pyrimidine ring was involved in the formation of a hydrogen bond with a noncrucial residue, Phe161. While the substituted benzene formed hydrophobic interactions with Val446 and Tyr472, which are not involved in the inhibition of the target protein. The lack of donor or acceptor functionalities at the side chain of compound **23** could be the reason of very few interactions with the crucial residues in the binding pocket, and ultimately responsible for the low inhibitory activity of this compound (Figure 9).

## 3. Conclusions

In conclusion, we have synthesized a series of 2-aminopyrimidine derivatives **1–27** and evaluated their *β*-glucuronidase inhibitory activity. Among them, compound **24** (IC_50_ = 2.8 ± 0.10 µM) showed an excellent activity, much superior than standard D-saccharic acid 1,4-lactone (IC_50_ = 45.75 ± 2.16 µM). Binding modes and molecular mechanics of ligands were also predicted *via* docking simulation studies and found that the presence of donor or acceptor functionalities is important for the potent inhibitory activity. Thus, current research has identified compound **24** as a potent *beta*-glucuronidase inhibitor with the potential to be studied further.

## 4. Experiment

### 4.1. General

NMR experiments were performed either on Bruker AM 300 or 400 MHz instruments (Bruker, Switzerland). CHN analyses were performed on a Carlo Erba Strumentazione Mod-1106, (Italy). Electron impact mass spectra (EI-MS) were recorded on a Finnigan MAT-311A (Germany). Thin-layer chromatography (TLC) was performed on precoated silica gel glass plates (Kieselgel 60, 254, E. Merck, Darmstadt, Germany) and visualized by either UV at 254 or 365 nm.

### 4.2. General Procedure for the Synthesis of 2-aminopyrimidine Derivatives

2-Aminopyrimidine derivatives **1–27** were synthesized by heating finely ground 2-amino-4,6-dichloropyrimidine (3 mmol), substituted amine (3 mmol), and triethylamine (6 mmol) in a solvent-free condition at 80–90 °C. The reaction was monitored by TLC using hexane and ethyl acetate as a solvent system. After completion, distilled water was added to the reaction mixture, and the precipitates obtained were filtered and crystallized using ethanol. In a few cases, where precipitates were not formed with the addition of water, the solvent (water) was dried under vacuum, and the resulting crude was purified by crystallization using ethanol.

#### 4.2.1. 6-Chloro-4-(*N*-phenyl)-2,4-pyrimidinediamine (**1**)

Brown solid (powder); yield: 83%; reaction time 5 h; *R_f_*: 0.46 (ethyl acetate/hexanes, 3:7); m.p. 175–177 °C; ^1^H-NMR (400 MHz, DMSO-*d_6_*): *δ* 9.29 (s, 1H, NH), 7.67 (d, *J*_2′,3′_ = *J*_6′,5′_ = 8.0 Hz, 2H, H-2′,6′), 7.30 (t, *J*_3′,2′/5′,6′_ *= J*_3′,4′/5′,4′_ = 7.6 Hz, 2H, H-3′, H-5′), 7.00 (t, *J*_4′,5′_ *= J*_4′,3′_ = 7.6 Hz, 1H, H-4′), 6.70 (s, 2H, NH_2_), 5.99 (s, 1H, H-5), ^13^C-NMR (100 MHz, DMSO-*d_6_*): *δ* 93.7 (C-5), 119.8 (C-2′, C-6′), 122.1 (C-3′, C-5′), 128.6 (C-4′), 139.9 (C-1′), 158.1 (C-4), 161.9 (C-2), 162.8 (C-6), EI-MS *m*/*z* (rel. int. %): 220.2 (M^+^, 95.5), 222.2 (M^+^ + 2, 29.1), 219.2 (100), 185.2 (6.8), 158.1 (9.7), 143.1 (26.4), 77.1 (13.9); Anal. Calcd for C_10_H_9_ClN_4_: C, 54.43; H, 4.11; N, 25.39; Found: C, 54.81; H, 4.00; N, 25.43.

#### 4.2.2. 6-Chloro-4-(*N*-(2-methoxy)phenyl)-2,4-pyrimidinediamine (**2**)

Brown solid (powder); yield: 84%; reaction time 4 h and thirty minutes; *R_f_*: 0.57 (ethyl acetate/hexanes, 4:6); m.p. 240–242 °C; ^1^H-NMR (400 MHz, DMSO-*d_6_*): *δ* 8.49 (s, 1H, NH), 8.01 (d, *J*_6′,5′_ = 8.0 Hz, 1H, H-6′), 7.05 (m, 2H, H-3′,4′), 6.92 (dt, *J*_5′,4′_ *= J*_5′,6′_ = 8.0 Hz, *J*_5′,3′_ = 2.4 Hz, 1H, H-5′), 6.58 (s, 2H, NH_2_), 6.13 (s, 1H, H-5), 3.81 (s, 3H, OCH_3_). EI-MS *m*/*z* (rel. int. %): 250.04 (M^+^, 11.2), 252 (M^+^ + 2, 3.7), 219 (100), 172 (6.2), 158 (11.6), 128 (6); Anal. Calcd for C_11_H_11_ClN_4_O: C, 52.70; H, 4.42; N, 22.35; Found: C, 51.01; H, 3.94; N, 21.63.

#### 4.2.3. 6-Chloro-4-(*N*-(3-methoxy)phenyl)-2,4-pyrimidinediamine (**3**)

Brown solid powder; yield: 82%; reaction time 6 h; *R_f_*: 0.55 (ethyl acetate/hexanes, 4:6); m.p. 178–180 °C; ^1^H-NMR (300 MHz, DMSO-*d_6_*): *δ* 9.27 (s, 1H, NH), 7.36 (s, 1H, H-2′), 7.18 (d, *J*_4′,5′_ *= J*_6′,5′_ = 5.1 Hz, 2H, H-4′,6′), 6.70 (s, 2H, NH_2_), 6.58 (m, 1H, H-5′), 5.99 (s, 1H, H-5), 3.74 (s, 3H, OCH_3_). EI-MS *m*/*z* (rel. int. %): 250.1 (M^+^, 77.8), 252.1 (M^+^ + 2, 25.9), 249 (82.4), 234 (9.4), 169 (100), 157 (56.9), 143 (63.5), 128 (82); Anal. Calcd for C_11_H_11_ClN_4_: C, 52.70; H, 4.42; N, 22.35; Found: C, 53.33; H, 4.35; N, 23.97.

#### 4.2.4. 6-Chloro-4-(*N*-(4-methoxy)phenyl)-2,4-pyrimidinediamine (**4**)

Dark yellow solid (powder); yield: 84%; reaction time 4 h; *R_f_*: 0.42 (ethyl acetate/hexanes, 4:6); m.p. 222–223 °C; ^1^H-NMR (300 MHz, DMSO-*d_6_*): *δ* 9.08 (s, 1H, NH), 7.51 (d, *J*_3′,2′_ *= J*_5′,6′_ = 9.0 Hz, 2H, H-3′,5′), 6.88 (d, *J*_2′,3′_ *= J*_6′,5′_ = 9.0 Hz, 2H, H-2′,6′), 6.58 (s, 2H, NH_2_), 5.88 (s, 1H, H-5), 3.72 (s, 3H, OCH_3_). EI-MS *m*/*z* (rel. int. %): 250.1 (M^+^, 100), 252.1 (M^+^ + 2, 30.8), 235.1 (53.6), 172 (5.9), 128 (9.7); Anal. Calcd for C_11_H_11_ClN_4_: C, 52.70; H, 4.42; N, 22.35; Found: C, 49.51; H, 3.94; N, 21.75.

#### 4.2.5. 6-Chloro-4-(*N*-(2,5-dimethoxy)phenyl)-2,4-pyrimidinediamine (**5**)

Brown solid (powder); yield: 85%; reaction time 5 h; *R_f_*: 0.59 (ethyl acetate/hexanes, 4:6); m.p. 182–184 °C; ^1^H-NMR (300 MHz, DMSO-*d_6_*): *δ* 8.48 (s, 1H, NH), 7.77 (s, 1H, H-6′), 6.94 (d, *J*_3′,4′_ = 8.7 Hz, 1H, H-3′), 6.63 (s, 2H, NH_2_), 6.59 (dd, *J*_4′,3′_ = 8.7 Hz, *J*_4′,6′_ = 3.0 Hz, 1H, H-4′), 6.22 (s, 1H, H-5), 3.76 (s, 3H, OCH_3_), 3.70 (s, 3H, OCH_3_). EI-MS *m*/*z* (rel. int. %): 280.2 (M^+^, 23.4), 282.2 (M^+^ + 2, 8.1), 265.1 (10.7), 249.1 (100); Anal. Calcd for C_12_H_13_ClN_4_O_2_: C, 51.34; H, 4.67; N, 19.96; Found: C, 51.72; H, 4.67; N, 19.72.

#### 4.2.6. 6-Chloro-4-(*N*-(3-methoxy-4-methyl)phenyl)-2,4-pyrimidinediamine (**6**)

Brown solid (powder); yield: 85%; reaction time 4 h; *R_f_*: 0.57 (ethyl acetate/hexanes, 3:7); m.p. 245–247 °C; ^1^H-NMR (300 MHz, DMSO-*d_6_*): *δ* 9.21 (s, 1H, NH), 7.32 (s, 1H, H-2′), 7.08 (dd, *J*_6′,5′_ = 8.1 Hz, *J*_6′,2′_ = 1.8 Hz, 1H, H-6′), 7.01 (d, *J*_5′,6′_ = 8.1 Hz, 1H, H-5′), 6.67 (s, 2H, NH_2_), 5.96 (s, 1H, H-5), 3.78 (s, 3H, OCH_3_), 2.07 (s, 3H, CH_3_). EI-MS *m*/*z* (rel. int. %): 264.2 (M^+^, 100), 266.1 (M^+^ + 2, 47.8), 249.1 (12.9), 221.1 (7.1), 187 (11.3); Anal. Calcd for C_12_H_13_ClN_4_O: C, 54.45; H, 4.95; N, 21.17; Found: C, 54.48; H, 4.38; N, 21.11.

#### 4.2.7. 6-Chloro-4-(*N*-(5-chloro-2,4-dimethoxy)phenyl)-2,4-pyrimidinediamine (**7**)

Yellow solid (powder); yield: 85%; reaction time 5 h; *R_f_*: 0.46 (ethyl acetate/hexanes, 4:6); m.p. 185–187 °C; ^1^H-NMR (300 MHz, DMSO-*d_6_*): *δ* 8.47 (s, 1H, NH), 7.84 (s, 1H, H-6′), 6.84 (s, 1H, H-3′), 6.58 (s, 2H, NH_2_), 5.99 (s, 1H, H-5), 3.87 (s, 3H, OCH_3_), 3.85 (s, 3H, OCH_3_). EI MS: *m*/*z* (rel. abund. %): 315 (M^+^, 10.6), 317 (M^+^ + 2, 7.0), 314 (63.0), 299 (37.6), 283 (100); Anal. Calcd for C_12_H_12_Cl_2_N_4_O_2_: C, 45.73; H, 3.34; N, 17.78; Found: C, 45.24; H, 3.12; N, 17.81. 

#### 4.2.8. 6-Chloro-4-(*N*-(4-n-butoxy)phenyl)-2,4-pyrimidinediamine (**8**)

Brown solid (powder); yield: 86%; reaction time 4 h; *R_f_*: 0.34 (ethyl acetate/hexanes, 3:7); m.p. 180–182 °C; ^1^H-NMR (300 MHz, DMSO-*d_6_*): *δ* 9.08 (s, 1H, NH), 7.48 (d, *J*_2′,3′_ *=J*_6′,5′_ = 9.0 Hz, 2H, H-2′,6′), 6.86 (d, *J*_3′,2′_ *= J*_5′,6′_ = 9.0 Hz, 2H, H-3′,5′), 6.59 (s, 2H, NH_2_), 5.88 (s, 1H, H-5), 3.93 (t, 2H, OCH_2_), 1.69 (t, *J* = 6.6 Hz, 2H, CH_2_), 1.46 (q, *J* = 15.3 Hz, *J* = 7.8 Hz, 2H, CH_3_), 0.94 (t, *J* = 7.2 Hz, 3H, CH_3_). EI-MS *m*/*z* (rel. int. %): 292.1 (M^+^, 76.3), 294.1 (M^+^ + 2, 25.1), 235 (100), 201 (9.2); Anal. Calcd for C_14_H_17_ClN_4_O: C, 57.44; H, 5.85; N, 19.14; Found: C, 58.48; H, 6.38; N, 19.11.

#### 4.2.9. 6-Chloro-4-(*N*-(4-n-octoxy)phenyl-2,4-pyrimidinediamine (**9**)

Brown solid (powder); yield: 85%; reaction time 4 h; *R_f_*: 0.48 (ethyl acetate/hexanes, 5:5); m.p. 185–187 °C; ^1^H-NMR (300 MHz, DMSO-*d_6_*): *δ* 9.08 (s, 1H, NH), 7.48 (d, *J*_3′,2′_ = *J*_5′,6′_ = 9.0 Hz, 2H, H-3′,5′) 6.86 (d, *J*_2′,3′_ = *J*_6′,5′_ = 9.0 Hz, 2H, H-2′,6′), 6.59 (s, 2H, NH_2_), 5.87 (s, 1H, H-5), 3.93 (t, 2H, OCH_2_(C_7_H_15_)), 1.69 (m, 2H, OCH_2_CH_2_(C_6_H_13_)), 1.38 (br.s, 10H, OC_2_H_4_(C_5_H_10_)CH_3_), 0.87 (t, 3H, O(C_7_H_14_)CH_3_); EI MS: *m*/*z* (rel. abund. %) 348 (M^+^, 68.2), 350 (M^+^ + 2, 22.6), 249 (58.2), 236 (91.6), 137 (100), 109 (68.9); Anal. Calcd for C_18_H_25_ClN_4_O: C, 61.97; H, 7.22; N, 16.06; Found: C, 63.30; H, 8.15; N, 16.00.

#### 4.2.10. 6-Chloro-4-(*N*-(4-bromo)phenyl)-2,4-pyrimidinediamine (**10**)

Yellow solid (powder); yield: 80%; reaction time 6 hours and thirty minutes; *R_f:_* 0.44 (ethyl acetate/hexanes, 3:7); m.p. 190–192 °C; ^1^H-NMR (300 MHz, DMSO-*d_6_*): *δ* 9.43 (s, 1H, NH), 7.68 (d, *J*_3′,2′_ *= J*_5′,6′_ = 6.9 Hz, 2H, H-3′,5′), 7.44 (d, *J*_2′,3′_ *= J*_6′,5′_ = 6.9 Hz, 2H, H-2′,6′), 6.77 (s, 2H, NH_2_), 5.99 (s, 1H, H-5). EI-MS *m*/*z* (rel. int. %): 299.1 (M^+^, 15.5), 301.1 (M^+^ + 2, 5.7), 298.1 (10), 171.1 (36.8), 158.1 (90), 128.1 (100); Anal. Calcd for C_10_H_8_BrCl: C, 40.10; H, 2.69; N, 18.70; Found: C, 35.71; H, 2.62; N, 14.52. 

#### 4.2.11. 6-Chloro-4-(*N*-(3-bromo)phenyl)-2,4-pyrimidinediamine (**11**)

Light yellow solid (powder); yield: 81%; reaction time 6 h; *R_f_*: 0.47 (ethyl acetate/hexanes, 3:7); m.p. 180–182 °C; ^1^H-NMR (300 MHz, DMSO-*d_6_*): *δ* 9.45 (s, 1H, NH), 7.93 (t, *J*_2′,4′_ *= J*_2′,6′_ = 1.8 Hz, 1H, H-2′), 7.68 (d, *J*_4′,5′_ = 8.7 Hz, 1H, H-4′), 7.25 (t, *J*_5′,4′_ *= J*_5′,6′_ = 8.1 Hz, 1H, H-5′), 7.15 (d, *J*_6′,5′_ = 8.1 Hz, 1H, H-6′), 6.82 (s, 2H, NH_2_), 5.99 (s, 1H, H-5). EI-MS *m*/*z* (rel. int. %): 299.2 (M^+^, 100), 301.2 (M^+^ + 2, 31.9), 298.2 (58.7); Anal. Calcd for C_10_H_8_BrCl: C, 40.10; H, 2.69; N, 18.70; Found: C, 40.96; H, 2.23; N, 18.60.

#### 4.2.12. 6-Chloro-4-(*N*-(4-chloro)phenyl)-2,4-pyrimidinediamine (**12**)

White solid (powder); yield: 78%; reaction time 14 h; *R_f_*: 0.46 (ethyl acetate/hexanes, 3:7); m.p. 182–184 °C; ^1^H-NMR (300 MHz, DMSO-*d_6_*): *δ* 9.42 (s, 1H, NH), 7.73 (d, *J*_3′,2′_ *= J*_5′,6′_ = 9.0 Hz, 2H, H-3′,5′), 7.32 (d, *J*_2′,3′_ *= J*_6′,5′_ = 9.0 Hz, 2H, H-2′,6′), 6.76 (s, 2H, NH_2_), 5.99 (s, 1H, H-5). EI-MS *m*/*z* (rel. int. %): 254.3 (M^+^, 100), 256.3 (M^+^ + 2, 65.7), 218.3 (4.8), 177.3 (24.6); Anal. Calcd for C_10_H_8_Cl_2_N_4_: C, 47.08; H, 3.16; N, 21.96; Found: C, 47.05; H, 2.82; N, 21.68.

#### 4.2.13. 6-Chloro-4-(*N*-(3-chloro)phenyl)-2,4-pyrimidinediamine (**13**)

Off white solid (powder); yield: 78%; reaction time 12 h; *R_f_*: 0.52 (ethyl acetate/hexanes, 4:6); m.p. 177–179 °C; ^1^H-NMR (300 MHz, DMSO-*d_6_*): *δ* 9.46 (s, 1H, NH), 7.87 (s, 1H, H-2′), 7.58 (dd, *J*_4′,5′_ = 8.1 Hz, *J*_4′,2′_ = *J*_4′,6′_ = 2.1 Hz,1H, H-4′), 7.31 (t, *J*_5′(4′_,_6′)_ = 8.1 Hz, 1H, H-5′), 7.02 (dd = *J*_6′,5′_ = 8.1 Hz, *J*_6′,4′_ = 2.1 Hz, 1H, H-6′), 6.82 (s, 2H, NH_2_), 5.99 (s, 1H, H-5); EI MS: *m*/*z* (rel. abund. %) 255 (M^+^, 83.9), 257 (M^+^ + 2, 20.7), 219 (15.6), 177 (50), 111 (26.7); Anal. Calcd for C_10_H_8_Cl_2_N_4_: C, 47.08; H, 3.16; N, 21.96; Found: C, 47.51; H, 3.94; N, 21.75.

#### 4.2.14. 6-Chloro-4-(*N*-(4-iodo)phenyl)-2,4-pyrimidinediamine (**14**)

Dark brown solid (powder); yield: 81%; reaction time 7 h; *R_f_*: 0.56 (ethyl acetate/hexanes, 3:7); m.p. 250–252 °C; ^1^H-NMR (300 MHz, DMSO-*d_6_*): *δ* 9.41 (s, 1H, NH), 7.59 (m, 4H, H-2′, 3′, 5′, 6′), 6.78 (s, 2H, NH_2_), 5.99 (s, 1H, H-5). EI-MS *m*/*z* (rel. int. %): 346 (M^+^, 100), 347.9 (M^+^ + 2, 33.0), 311 (4.4), 219 (17.6), 142 (7.1); Anal. Calcd for C_10_H_8_ClN_4_I: C, 34.66; H, 2.33; N, 16.17; Found: C, 38.05; H, 2.07; N, 17.12.

#### 4.2.15. 6-Chloro-4-(*N*-benzyl)-2,4-pyrimidinediamine (**15**)

Light yellow solid (powder); yield: 88%; reaction time 4 h; *R_f_*: 0.48 (ethyl acetate/hexanes, 3:7); m.p. 255–257 °C; ^1^H-NMR (300 MHz, DMSO-*d_6_*): *δ* 7.61 (s, 1H, NH), 7.34 (m, 5H, H-2′,3′,4′,5′,6′), 6.42 (s, 2H, NH_2_), 5.77 (s, 1H, H-5), 4.45 (s, 2H, CH_2_); EI MS: *m*/*z* (rel. abund. %) 234 (M^+^, 100), 236 (M^+^ + 2, 34.1), 157 (10.8), 106 (85.9), 91 (45.1); Anal. Calcd for C_11_H_11_ClN_4_: C, 56.30; H, 4.72; N, 23.87; Found: C, 56.45; H, 4.46; N, 21.76. 

#### 4.2.16. 6-Chloro-4-(*N*-(2-isopropyl)phenyl)-2,4-pyrimidinediamine (**16**)

Light yellow solid (powder); yield: 78%; reaction time 10 h; *R_f_*: 0.53 (ethyl acetate/hexanes, 3:7); m.p. 170–172 °C; ^1^H-NMR (300 MHz, DMSO-*d_6_*): *δ* 8.71 (s, 1H, NH), 7.35 (d, *J*_3′,5′_ = 6.9 Hz, 1H, H-3′), 7.20 (br.s, 3H, H-4′,5′,6′), 6.48 (s, 2H, NH_2_), 5.65 (s, 1H, H-5), 3.12 (m, 1H, CH(CH_3_)_2_), 1.13 (d, 6H, CH(CH_3_)_2_). EI-MS *m*/*z* (rel. int. %): 262.10 (M^+^, 96.1), 264.2 (M^+^ + 2, 31.7), 247.2 (38.4), 219.1 (100), 169.2 (45.2), 120.2 (64.2); Anal. Calcd for C_13_H_15_ClN_4_: C, 59.43; H, 5.75; N, 21.32; Found: C, 57.29; H, 5.69; N, 21.59.

#### 4.2.17. 6-Chloro-4-(*N*-(2,3-dimethyl)phenyl)-2,4-pyrimidinediamine (**17**)

Off white solid (powder); yield: 81%; reaction time 5 h; *R_f_*: 0.53 (ethyl acetate/hexanes, 4:6); m.p. 176–178 °C; ^1^H-NMR (300 MHz, DMSO-*d_6_*): *δ* 8.72 (s, 1H, NH), 7.08 (m, 3H, H-4′,5′,6′), 6.47 (s, 2H, NH_2_), 5.65 (s, 1H, H-5), 2.25 (s, 3H, CH_3_), 2.04 (s, 3H, CH_3_). EI-MS *m*/*z* (rel. int. %): 248 (M^+^, 100), 250 (M^+^ + 2, 60.7), 232 (19.7), 170 (97.7), 119 (4.1); Anal. Calcd for C_12_H_13_ClN_4_: C, 57.95; H, 5.27; N, 22.53; Found: C, 54.34; H, 4.85; N, 21.28.

#### 4.2.18. 6-Chloro-4-(*N*-(2,5-dimethyl)phenyl)-2,4-pyrimidinediamine (**18**)

Light brown solid (powder); yield: 81%; reaction time 5 h; *R_f_*: 0.56 (ethyl acetate/hexanes, 3:7); m.p. 173–175 °C; ^1^H-NMR (400 MHz, DMSO-*d_6_*): *δ* 8.60 (s, 1H, NH), 7.13 (m, 2H, H-3′,6′), 6.93 (d, *J*_4′,5′_ = 7.6 Hz, 1H, H-4′), 6.47 (s, 2H, NH_2_), 5.72 (s, 1H, H-5), 2.25 (s, 3H, CH_3_), 2.11 (s, 3H, CH_3_). EI-MS *m*/*z* (rel. int. %): 248.1 (M^+^, 100), 250.1 (M^+^ + 2, 44.2), 213 (16.7), 169 (13.2), 119 (2.0); Anal. Calcd for C_12_H_13_ClN_4_: C, 57.95; H, 5.27; N, 22.53; Found: C, 58.22; H, 5.34; N, 22.40.

#### 4.2.19. 6-Chloro-4-(*N*-(5-chloro-2-methyl)phenyl)-2,4-pyrimidinediamine (**19**)

Off white solid (powder); yield: 79%; reaction time 4 h and thirty minutes; *R_f_*: 0.51 (ethyl acetate/hexanes, 4:6); m.p. 175–177 °C; ^1^H-NMR (300 MHz, DMSO-*d_6_*): *δ* 8.68 (s, 1H, NH), 7.57 (d, *J*_6′,4′_ = 2.1 Hz, 1H, H-6′), 7.25 (d, *J*_3′,4′_ = 8.1 Hz, 1H, H-3′), 7.13 (dd, *J*_4′,3′_ = 8.1 Hz, *J*_4′,6′_ = 2.1 Hz, 1H, H-4′), 6.60 (s, 2H, NH_2_), 5.94 (s, 1H, H-5), 2.17 (s, 3H, CH_3_); EI MS: *m*/*z* (rel. abund. %) 268 (M^+^, 100), 270 (M^+^ + 2, 68.7), 253 (89.8), 233 (31.1), 140 (36.3), 125 (14.2); Anal. Calcd for C_11_H_10_Cl_2_N_4_: C, 49.09; H, 3.75; N, 20.82; Found: C, 49.51; H, 3.91; N, 20.70.

#### 4.2.20. 6-Chloro-4-(*N*-(2,4-dimethyl)phenyl)-2,4-pyrimidinediamine (**20**)

Brown solid (powder); yield: 80%; reaction time 5 h and thirty minutes; *R_f_*: 0.55 (ethyl acetate/hexanes, 4:6); m.p. 178–180 °C; ^1^H-NMR (400 MHz, DMSO-*d_6_*): *δ* 8.58 (s, 1H, NH), 7.17 (d, *J*_5′,6′_ = 7.6 Hz, 1H, H-5′), 7.05 (s, 1H, H-3′), 6.99 (d, *J*_6′,5′_ = 7.6 Hz, 1H, H-6′), 6.44 (s, 2H, NH_2_), 5.68 (s, 1H, H-5). EI-MS *m*/*z* (rel. int. %): 248 (M^+^, 100), 250 (M^+^ + 2, 45.5), 247 (60.9), 233 (93.3), 213 (27.9), 170 (64.5), 149 (56.0); Anal. Calcd for C_12_H_13_ClN_4_: C, 57.95; H, 5.27; N, 22.53; Found: C, 57.23; H, 5.32; N, 21.58.

#### 4.2.21. 6-Chloro-4-(*N*-(3,4-dimethyl)phenyl)-2,4-pyrimidinediamine (**21**)

Off white solid (powder); yield: 84%; reaction time 4 h and thirty minutes; *R_f_*: 0.58 (ethyl acetate/hexanes, 3:7); m.p. 179–180 °C; ^1^H-NMR (300 MHz, DMSO-*d_6_*): *δ* 9.10 (s, 1H, NH), 7.39 (d, *J*_5′,6′_ = 9.3 Hz, 2H, H-5′,2′), 7.04 (d, *J*_6′,5′_ = 8.1 Hz, 1H, H-6′), 6.64 (s, 2H, NH_2_), 5.93 (s, 1H, H-5), 2.19 (s, 3H, CH_3_), 2.15 (s, 3H, CH_3_). EI-MS *m*/*z* (rel. int. %): 248.2 (M^+^, 100), 250.2 (M^+^ + 2, 35.9), 247.2 (95.8), 233. (4.3), 213.1 (8.7), 171.2 (39.2), 128.1 (9.9); Anal. Calcd for C_12_H_13_ClN_4_: C, 57.95; H, 5.27; N, 22.53; Found: C, 57.29; H, 5.69; N, 22.59.

#### 4.2.22. 6-Chloro-4-(*N*-(4-ethyl)phenyl)-2,4-pyrimidinediamine (**22**)

Brown solid (powder); yield: 84%; reaction time 7 h; *R_f_*: 0.47 (ethyl acetate/hexanes, 3:7); m.p. 172–174 °C; ^1^H-NMR (400 MHz, DMSO-*d_6_*): *δ* 9.19 (s, 1H, NH), 7.53 (d, *J*_3′,2′_ *= J*_5′,6′_ = 8.4 Hz, 2H, H-3′,5′), 7.12 (d, *J*_2′,3′_ *= J*_6′,5′_ = 8.4 Hz, 2H, H-2′, H-6′), 6.64 (s, 2H, NH_2_), 5.95 (s, 1H, H-5), 2.57 (q, 2H, CH_2_), 1.17 (t, 3H, CH_3_). EI-MS *m*/*z* (rel. int. %): 248.1 (M^+^, 100), 250.1 (M^+^ + 2, 27.6), 233.1 (100); Anal. Calcd for C_12_H_13_ClN_4_: C, 57.95; H, 5.27; N, 22.53; Found: C, 58.86; H, 5.36; N, 22.68.

#### 4.2.23. 6-Chloro-4-(*N*-(4-n-butyl)phenyl)-2,4-pyrimidinediamine (**23**)

White solid (powder); yield: 82%; reaction time 5 h; *R_f_*: 0.50 (ethyl acetate/hexanes, 3:7); m.p. 175–177 °C; ^1^H-NMR (300 MHz, CDCl_3_): *δ* 7.24 (s, 1H, NH), 6.55 (br.s, 2H, H-3′,5′), 6.04 (br.s, 2H, H-2′,6′), 4.87 (s, 2H, NH_2_), 2.61 (t, 2H, CH_2_), 1.62 (m, 2H, CH_2_), 1.39 (m, 2H, CH_2_), 0.94 (t, 3H, CH_3_). EI-MS *m*/*z* (rel. int. %): 276.1 (M^+^, 21.5), 278.2 (M^+^ + 2, 7.4), 233 (100), 155 (25.9), 106 (69.4); Anal. Calcd for C_14_H_17_ClN_4_: C, 60.76; H, 6.19; N, 20.24; Found: C, 61.85; H, 6.32; N, 20.63.

#### 4.2.24. 4-Chloro-6-(1-piperazinyl)-2-pyrimidinylamine (**24**)

Dark yellow solid (powder); yield: 82%; reaction time 6 h; *R_f_*: 0.36 (dichloromethane/methanol, 9:1); m.p. >300 °C; ^1^H-NMR (300 MHz, DMSO-*d_6_*): *δ* 6.44 (s, 2H, NH_2_), 6.05 (s, 1H, H-5), 3.49 (m, 5H, NH, 2×H-3′, H-5′), 2.74 (m, 4H, 2×H-2′, H-6′), ^13^C-NMR (100 MHz, DMSO-*d_6_*): *δ* 43.8 (C-3′, C-5′), 44.6 (C-2′, C-6′), 90.6 (C-5), 159.4 (C-4), 162.4 (C-2), 163.2 (C-6). EI-MS *m*/*z* (rel. int. %): 213.1 (M^+^, 35.9), 215.1 (M^+^ + 2, 11.9), 171.1 (97), 157 (52.7), 145 (100), 129 (27.2); Anal. Calcd for C_8_H_12_ClN_5_: C, 44.97; H, 5.66; N, 32.78; Found: C, 45.51; H, 5.94; N, 31.75.

#### 4.2.25. 6-Chloro-6-(4-phenyl-1-piprazinyl)-2-pyrimidine amine (**25**)

Light yellow solid (powder); yield: 82%; reaction time 7 h; *R_f_*: 0.56 (ethyl acetate/hexanes, 3:7); m.p. 200–202 °C; ^1^H-NMR (300 MHz, DMSO-*d_6_*): *δ* 7.25 (d, *J*_3″,2″_ *= J*_5″,6″_ = 8.4 Hz, 2H, H-3″, H-5″), 6.98 (d, *J*_2″,3″_ *= J*_6″,5″_ = 8.4 Hz, 2H, H-2″, H-6″), 6.82 (t, *J*_4″,5″_ *= J*_4″,3″_ = 7.2 Hz, 1H, H-4″), 6.52 (s, 2H, NH_2_), 6.15 (s, 1H, CH), 3.75 (br.s, 4H, 2 × H-2′, H-6′), 3.17 (br.s, 4H, 2×H-3′, H-5′). EI-MS *m*/*z* (rel. int. %): 289 (M^+^, 74.9), 291 (M^+^ + 2, 23.0), 212.2 (3.2), 157.0 (100), 77.1 (17.7); Anal. Calcd for C_14_H_16_ClN_5_: C, 58.03; H, 5.57; N, 24.17; Found: C, 52.59; H, 4.82; N, 21.75.

#### 4.2.26. 6-Chloro-4-(*N*-(3-pyridinyl)methyl)-2,4-pyrimidinediamine (**26**)

Dark yellow solid (powder); yield: 87%; reaction time 5 h; *R_f_*: 0.48 (dichloromethane/methanol, 9:1); m.p. 210–212 °C; ^1^H-NMR (300 MHz, DMSO-*d_6_*): *δ* 8.52 (s, 1H, NH), 8.45 (dd, *J*_4′,5′_ = 4.8 Hz, *J*_4′,6′_ = 1.5 Hz, 1H, H-4′), 7.70 (m, 2H, H-2′, H-5′),7.36 (m, 1H, H-6′), 6.46 (s, 2H, NH_2_), 5.78 (s, 1H, CH), 4.47 (d, 2H, *J =* 4.8 Hz CH_2_). EI-MS *m*/*z* (rel. int. %): 235.1 (M^+^, 100), 237.1 (M^+^ + 2, 36.1), 200.1 (36), 157.1 (23), 129.0 (12.6), 92.1 (28.9); Anal. Calcd for C_10_H_10_ClN_5_: C, 50.96; H, 4.28; N, 29.72; Found: C, 51.48; H, 4.16; N, 29.90.

#### 4.2.27. 6-Chloro-4-(*N*-(furan-2-yl)methyl)-2,4-pyrimidinediamine (**27**)

Brown solid (powder); yield: 86%; reaction time 4 h; *R_f_*: 0.42 (ethyl acetate/hexanes, 4:6); m.p. 140–142 °C; ^1^H-NMR (300 MHz, DMSO-*d_6_*): *δ* 7.57 (s, 1H, NH), 7.52 (br.s, 1H, H-5′), 6.46 (s, 2H, NH_2_), 6.39 (m, 2H, H-3′, H-4′), 5.78 (s, 1H, H-5), 4.43 (s, 2H, CH_2_). EI MS: *m*/*z* (rel. abund. %) 224 (M^+^, 100), 226 (M^+^ + 2, 30.5), 195 (62.1), 128 (32.4); Anal. Calcd for C_9_H_9_ClN_4_O: C, 48.12; H, 4.04; N, 24.94; Found: C, 49.33; H, 3.93; N, 25.41.

### 4.3. Protocol for β-Glucuronidase Inhibition

2-Aminopyrimidine derivatives **1–27** were evaluated for their *β*-glucuronidase inhibition by using a literature protocol [34]. *β*-Glucuronidase inhibitory activity of compounds **1–27** was measured by observing absorbance of *p*-nitrophenol (hydrolyzed from *p*-nitrophenyl-*b*-D-glucuronide (N-1627) at 405 nm using spectrophotometer.

The reaction mixture comprising of 0.1 M acetate buffer (185 µL), test compound solution (5 µL, 100% DMSO was used to dissolve test compound) and 1 U/well or 1 U/250 µL enzyme solution (10 µL, above mentioned buffer was used to dissolve the enzyme) was incubated for thirty minutes at 37 °C. Then 50 µL of 0.4 mM *p*-nitrophenyl-*β*-D-glucuronide was added in each well, and plates were read on a multiple reader (SpectraMax plus 384, Molecular Devices, San Jose, CA, USA), at 405 nm. Whole procedure was repeated three times for each compound. D-saccharic acid 1,4-lactone was used as the standard inhibitor of *ß*-glucuronidase.

### 4.4. Protocol for Urease Inhibition

The 2-aminopyrimidine derivatives **1–27** were evaluated for their in vitro urease inhibition potential by using literature protocol [35]. Urease inhibitory activity of compounds **1–27** was determined by measuring ammonia evolution *via* indophenol method.

The reaction mixture comprising of Jack bean urease enzyme (25 μL), buffer solution of PH 6.8 (55 μL), and urea (100 mM) was incubated for fifteen minutes at 30 °C in a 96-well plate. Then, in each well, 45 μL phenol reagent (1% *w*/*v* phenol and 0.005% *w*/*v* sodium nitroprussside) and 70 μL alkali reagent (0.5% *w*/*v* NaOH and 0.1% active chloride NaOCl) were added. Total volume was maintained at 200 μL. After fifty minutes absorbance was measured at 630 nm with the help of a microplate reader (Molecular Devices, USA). All assay was repeated thrice with final volume 200 μL for each compound. Percentage inhibitions were calculated from the formula {100−(OD_testwell_/OD_control_) × 100}. Thiourea was used as the standard inhibitor of urease.

### 4.5. Protocol for DPPH Radical Scavenging

The 2-aminopyrimidine derivatives **1–27** were evaluated for their DPPH radical scavenging activity by using a literature protocol [36]. Compounds **1–27** (0.2 mM, 100% DMSO was used to make the solution) and stable free radical DPPH (300 μM, ethanol was used to make the solution) were incubated for thirty minutes at 37 °C. Then a decrease in absorbance was observed at 515 nm using multi-plate reader (Spectra MAX-340). The %Radical scavenging activity was calculated by the formula %RSA= 100 − {(OD test compound/OD control) × 100}.

### 4.6. Protocol for Superoxide Scavenging

Compounds **1–27** were evaluated for their superoxide scavenging activity by the modified method used by Ferda [37]. The reaction mixture comprised of 40 µL of 280 µM β-nicotinamide adenine dinucleotide reduced form (NADH), 40 µL of 80 µM nitro blue tetrazolium (NBT), 20 µL of 8 µM phenazine methosulphate (PMS), 10 µL of 1 mM sample and 90 µL of 0.1 M phosphate buffer (pH 7.4). The reagents were prepared in buffer and sample in DMSO. The reaction was performed in a 96-well microtiter plate at room temperature and absorbance was measured at 560 nm. The formation of superoxide was monitored by measuring the formation of water-soluble blue Formazan dye. A lower absorbance of reaction mixture indicates a higher scavenging activity of the sample. Percent radical scavenging activity (%RSA) of samples was determined in comparison with a control using formula % RSA = 100 − {(OD test compound/OD control) × 100}.

### 4.7. Molecular Docking Protocol

The 3-D structures of 2-aminopyrimidine derivatives were sketched by MOE 2013, followed by protonation, minimization, and charge application of these compounds. A 3-D crystal structure of the target protein, i.e., *β*-glucuronidase from *E. coli*, was obtained from the protein data bank which is a homodimer comprising of chains A and B. Chain A was extracted and taken for further preparation involving the addition of missing atoms and correction of bonds and angles *via* the auto-correction tool in MOE. Furthermore, the protein was protonated, charged, and minimized to carry out the docking simulation. Active site information was obtained from the literature and utilized to focus on the key residues involved in the inhibition of the target enzyme [33]. The default docking parameters, including the Triangle matcher algorithm with two rescoring functions (London dG and GBVI/WSA dG), were specified for docking simulation of newly synthesized 2-aminopyrimidine derivatives. A total of 30 conformations of each compound were generated to predict their best possible binding pose. Finally, the docking results were grouped in output files in mdb format. This output file was further visually inspected to evaluate the key protein-ligand interactions, responsible for the inhibitory activity of 2-aminopyrimidine derivatives, within the active site of the target protein.

## Figures and Tables

**Table 1:** List of synthesized compounds **1–27** and their in vitro *β*-glucuronidase inhibition activity.

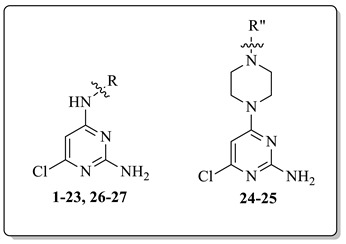					
**Comp. No.**	**R**	**IC_50_ ± SEM (µM)**	**Comp. No.**	**R/R″**	**IC_50_ ± SEM (µM)**
**1**	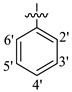	NA	**15**	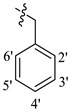	NA
**2**	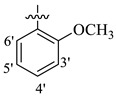	NA	**16**	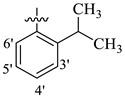	NA
**3**	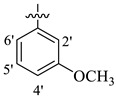	NA	**17**	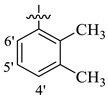	NA
**4**	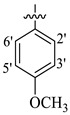	NA	**18**	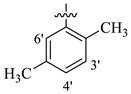	NA
**5**	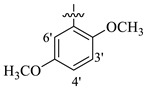	NA	**19**	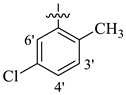	NA
**6**	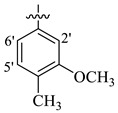	NA	**20**	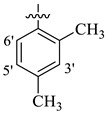	NA
**7**	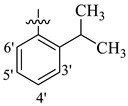	NA	**21**	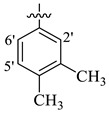	NA
**8**	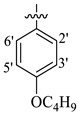	72.0 ± 6.20	**22**	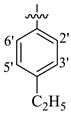	300.24 ± 12.15
**9**	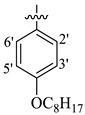	126.43 ± 6.16	**23**	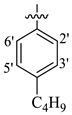	257.0 ± 4.18
**10**	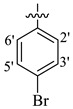	NA	**24**	H	2.8 ± 0.10
**11**	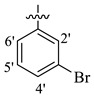	NA	**25**	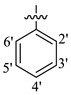	NA
**12**	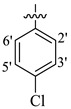	NA	**26**	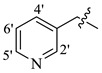	NA
**13**	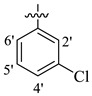	NA	**27**	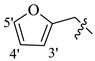	NA
**14**	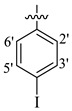	NA		D-Saccharic acid 1,4-lactone	45.75 ± 2.16

SEM is the standard error of the mean; NA Not active; D-saccharic acid 1,4-lactone is standard inhibitor used for *β*-glucuronidase study.

**Table 2:** Classification of all synthesized compounds **1–27**.

Groups	IC_50_	Compound
**Group A** (**Highly active**)	1–50 µM	Compound **24**
**Group B** (**Moderately active**)	50–100 µM	Compound **8**
**Group C** (**Least active**)	≥100 µM	Compounds **9**, **22**, **23**
**Group D** (**Inactive**)	No inhibition	Compounds **1–7**, **10–21**, and **25–27**

## Data Availability

Additional details of experiments and data may be asked *via* email to the corresponding authors.

## Sample Availability

Samples of the compounds **1–27** are available from the authors.
